# Supplementary material for: Design and Validation of a Simplified Method to Determine Minimum Bactericidal Concentration in Nontuberculous Mycobacteria
Source: Antibiotics (Basel). 2025 Apr 4;14(4):381. doi: 10.3390/antibiotics14040381 (PMC12023921; doi:10.3390/antibiotics14040381)
Supplement: Supplementary file 1 [file antibiotics-14-00381-s001.zip › antibiotics-3543130-supplementary.pdf]

Table S1. The MIC_{50}, MIC_{90} and MBC_{50} and MBC_{90} values of the three SGM species for the 13 antibiotics included in the SLOMYCOI Sensititre™ Thermo Scientific™ microtiter plate.

*: Total number of isolates analysed for each antibiotic. Only were included the isolates having MIC between the rang of each antibiotic in the Sensititre™ panel.

	M. intracellulare complex (N=99)					*M. avium* (N=64)					*M. xenopi* (N=17)				
**Antibiotic**	**Nº isolates***	**MIC_{50}**	**MIC_{90}**	**MBC_{50}**	**MBC_{90}**	**Nº isolates***	**MIC_{50}**	**MIC_{90}**	**MBC_{50}**	**MBC_{90}**	**Nº isolates***	**MIC_{50}**	**MIC_{90}**	**MBC_{50}**	**MBC_{90}**
**AMK**	98	8	16	8	16	64	8	16	16	32	17	2	8	2	8
**CIP**	86	8	16	16	>16	55	8	16	16	>16	17	1	4	1	4
**CLA**	98	2	4	4	8	62	4	8	8	16	14	0.12	1	0.25	1
**DOX**	11	16	16	>16	>16	6	16	16	>16	>16	3	16	16	>16	>16
**EMB**	87	8	16	16	>16	58	8	16	16	>16	12	4	16	8	>16
**ETH**	57	2.5	20	20	>20	60	2.5	10	20	>20	15	1.2	5	5	>20
**INH**	63	4	8	8	>8	48	8	8	>8	>8	15	0.5	4	2	>8
**LIN**	94	32	32	32	64	62	32	64	64	>64	17	4	16	4	16
**MOX**	99	2	2	2	4	63	2	4	4	>8	16	0.12	1	0.5	1
**RFB**	98	0.5	1	1	4	57	1	2	2	4	13	0.5	1	0.5	2
**RIF**	83	2	8	8	>8	39	4	8	>8	>8	15	0.5	2	1	4
**STR**	81	16	32	32	>64	53	32	64	64	>64	17	2	16	2	64
**SXT**	37	4	8	8	>8	29	4	8	>8	>8	11	0.25	2	2	8

MIC: minimum inhibitory concentration, MBC: minimum bactericidal concentration, AMK: amikacin, CIP: ciprofloxacin, CLA: clarithromycin, DOX: doxycycline, EMB: ethambutol, ETH: ethionamide, INH: isoniazid, LIN: linezolid, MOX: moxifloxacin, RFB: rifabutin, RIF: rifampicin, STR: streptomycin, SXT: cotrimoxazole.

Table S2. The MIC_{50}, MIC_{90} and MBC_{50} and MBC_{90} values of the three RGM species for the 15 antibiotics included in the RAPMYCOI Sensititre™ Thermo Scientific™ microtiter plate.

*: Total number of isolates analysed for each antibiotic. Only were included the isolates having MIC between the rang of each antibiotic in the Sensititre™ panel. (-): no isolates included. MIC: minimum inhibitory concentration, MBC: minimum bactericidal concentration, AMK: amikacin, AUG: amoxicillin clavulanic acid, AXO: ceftriaxone, CIP:

	M. abscessus (N=29)					*M. chelonae* (N=13)					*M. fortuitum* (N=7)				
**Antibiotic**	**Nº isolates***	**MIC_{50}**	**MIC_{90}**	**MBC_{50}**	**MBC_{90}**	**Nº isolates***	**MIC_{50}**	**MIC_{90}**	**MBC_{50}**	**MBC_{90}**	**Nº isolates***	**MIC_{50}**	**MIC_{90}**	**MBC_{50}**	**MBC_{90}**
**AMK**	27	8	16	32	>64	12	16	64	32	>64	7	2	2	4	16
**AUG**	9	64	64	>64	>64	2	64	64	>64	>64	6	16	32	>64	>64
**AXO**	6	64	64	>64	>64	2	4	64	32	>64	0	-*	-	-	-
**CIP**	13	4	4	>4	>4	10	2	4	4	>4	7	0.25	1	0.5	>4
**CLA**	NA	NA	NA	NA	NA	13	0.25	2	0.5	2	7	0.5	8	>16	>16
**DOX**	7	16	16	>16	>16	2	0.12	0.25	8	>16	7	0.25	16	2	>16
**FEP**	9	32	32	>32	>32	7	32	32	>32	>32	1	32	32	>32	>32
**FOX**	27	64	128	128	>128	4	128	128	>128	>128	7	32	64	>128	>128
**IMI**	25	16	32	>64	>64	9	8	64	>64	>64	7	4	8	>64	>64
**LIN**	26	16	32	>32	>32	13	8	32	32	>32	5	4	8	>32	>32
**MIN**	5	8	8	>8	>8	1	1	1	8	8	7	1	8	8	>8
**MOX**	19	8	8	>8	>8	12	4	8	8	>8	7	0.5	1	1	>8
**STX**	10	8	8	>8	>8	7	8	8	>8	>8	7	4	8	>8	>8
**TGC**	29	0.25	1	4	>4	13	0.5	1	4	>4	7	0.25	0.5	1	2
**TBR**	23	8	16	>16	>16	13	2	8	4	8	5	16	16	16	>16

ciprofloxacin, CLA: clarithromycin, DOX: doxycycline, FEP: cefepime, FOX: cefoxitin, IMI: imipenem, LIN: linezolid, MIN: minocycline, MOX: moxifloxacin, SXT: cotrimoxazole, TGC: tigecycline, TBR: tobramycin. NA: data not available. *M. abscessus*: AMK (27), TGC (29); *M.chelonae*: CLA (13), DOX (2), MIN (1), TGC (13); *M. fortuitum*: AMK (7), AUG (6), CIP (7), CLA (7), FOX (7), IMI (7), LIN (7), MOX (7), TGC (7).

Table S3: Comparison of commonly used minimum bactericidal concentration determination methods with the method used by our group (Reincubation).

Method	Description	Advantages	Disadvantage
**Reincubation**	MIC plate is reincubated, and and MBC is determined by visual bacterial growth as + or -.	• Simplified protocol<br>• Faster results<br>• Low workload	
**Broth Microdilution or broth Macrodilution + CFU counting onto agar [11]**	Content from MIC plates or tubes is transferred onto agar plates and CFUs are counted.	• Provides quantitative data on the MBC<br>• Widely accepted<br>• Clear results<br>• Clear endpoints<br>• Reliable	Labor intensive<br>Time consuming
**Broth Microdilution + liquid subculture (+/-) [14-27]**	Content from MIC plates is transferred to fresh liquid medium and MBC is determined by visual bacterial growth as + or -.	• Provides quantitative data on the MBC.<br>• Widely accepted<br>• Clear results<br>• Clear endpoints<br>• Reliable	Labor intensive<br>Time consuming

CFU: colony-forming unit, MIC: minimum inhibitory concentration, MBC: minimum bactericidal concentration.
